# Long-term feeding with high plant protein based diets in gilthead seabream (*Sparus aurata*, L.) leads to changes in the inflammatory and immune related gene expression at intestinal level

**DOI:** 10.1186/s12917-018-1626-6

**Published:** 2018-10-03

**Authors:** Guillem Estruch, Maria Carmen Collado, Raquel Monge-Ortiz, Ana Tomás-Vidal, Miguel Jover-Cerdá, David S Peñaranda, Gaspar Pérez Martínez, Silvia Martínez-Llorens

**Affiliations:** 1 Aquaculture and Biodiversity Research Group, Institute of Science and Animal Technology, (ICTA), Universitat Politècnica de València, Camino de Vera s/n, 46022 Valencia, Spain; 2 Institute of Agrochemistry and Food Technology, Department of Biotechnology, Spanish National Research Council (IATA-CSIC), Av. Agustin Escardino 7, 46980 Paterna, Spain

**Keywords:** Gilthead seabream, Vegetable meal, Squid meal, Krill meal, Intestine, Histology, Gene expression

## Abstract

**Background:**

In order to ensure sustainability of aquaculture production of carnivourous fish species such as the *gilthead seabream* (*Sparus aurata*, L.), the impact of the inclusion of alternative protein sources to fishmeal, including plants, has been assessed. With the aim of evaluating long-term effects of vegetable diets on growth and intestinal status of the on-growing gilthead seabream (initial weight = 129 g), three experimental diets were tested: a strict plant protein-based diet (VM), a fishmeal based diet (FM) and a plant protein-based diet with 15% of marine ingredients (squid and krill meal) alternative to fishmeal (VM+). Intestines were sampled after 154 days. Besides studying growth parameters and survival, the gene expression related to inflammatory response, immune system, epithelia integrity and digestive process was analysed in the foregut and hindgut sections, as well as different histological parameters in the foregut.

**Results:**

There were no differences in growth performance (*p* = 0.2703) and feed utilization (*p* = 0.1536), although a greater fish mortality was recorded in the VM group (*p* = 0.0141). In addition, this group reported a lower expression in genes related to pro-inflammatory response, as Interleukine-1β (*il1β*, *p* = 0.0415), Interleukine-6 (*il6, p* = 0.0347) and cyclooxigenase-2 (*cox2*, *p* = 0.0014), immune-related genes as immunoglobulin M (*igm, p* = 0.0002) or bacterial defence genes as alkaline phosphatase (*alp*, *p* = 0.0069). In contrast, the VM+ group yielded similar survival rate to FM (*p* = 0.0141) and the gene expression patterns indicated a greater induction of the inflammatory and immune markers (*il1β*, *cox2* and *igm*). However, major histological changes in gut were not detected.

**Conclusions:**

Using plants as the unique source of protein on a long term basis, replacing fishmeal in aqua feeds for gilthead seabream, may have been the reason of a decrease in the level of different pro-inflammatory mediators (il1 *β, il6 and cox2)* and immune-related molecules (*igm* and *alp*), which reflects a possible lack of local immune response at the intestinal mucosa, explaining the higher mortality observed. Krill and squid meal inclusion in vegetable diets, even at low concentrations, provided an improvement in nutrition and survival parameters compared to strictly plant protein based diets as VM, maybe explained by the maintenance of an effective immune response throughout the assay.

**Electronic supplementary material:**

The online version of this article (10.1186/s12917-018-1626-6) contains supplementary material, which is available to authorized users.

## Background

Fishmeal replacement in feeds is one of the main challenges in aquaculture farming in order to ensure the sustainability of the production of aquaculture species, especially in carnivorous species [1]. Plant sources have been used as substitutes in order to reduce the use of fishmeal [2] and to develop more economical and environmentally sustainable feeds compared to fishmeal based diets [1, 3].

Tolerance to vegetable products depends on species [4]. In the case of gilthead seabream, although high or total replacements of fishmeal by vegetable meal have been successfully achieved in terms of growth [5, 6], detrimental effects on nutrient digestibility and absorption [7, 8] have also been reported. Moreover, histomorphological gut and liver alterations [4, 9–11]**,** immune status disorders [9] or gut microbial imbalances [12] have been described. Thus, the use of certain agricultural by-products seems to ultimately lead to a lower feed conversion efficiency and an increase in both the susceptibility against diseases and bacterial and parasitic infections [13], which may be induced by an immune deficiency status or disruptions on the inflammatory response.

Hence, dietary and nutritional factors have a great influence on growth and immune response of fish [14]. Among other physiological processes, fish gut particularly plays a key role in the digestion and absorption of nutrients, in the immune response to potential pathogenic invasions and in the protection against environmental stressors [15]. The intestinal status in response to dietary changes has been widely assessed in fish, including gilthead seabream [16–21]. In particular, the impact of lowfishmeal diets on the intestinal physiology of different species has been assessed in different stages of the growing phase [22, 23].

A wide set of physiological parameters can be evaluated by using different techniques. Gene expression approaches allow to analyse different genes involved in different processes [24] including digestion (digestive enzymes, nutrient transporters), epithelial structure, inflammatory processes (cytokines and other proinflammatory mediators), and innate and adaptive immune response (mucins, genes codifying for antibodies), obtaining a snapshot of the whole response that can indeed provide hints and new insights to dietary impact on the intestinal status. On the other hand, histological assessment of the different gut layers can provide some valuable information on possible inflammatory reactions, as well as morphological adaptations to face with the dietary modifications [25].

In addition to detrimental effects associated to anti-nutritional factors [26, 27], whose impact depends on the tolerance of different species, fishmeal substitutions by great proportions of vegetable meals in fish diet could result in amino acid imbalances and palatability problems [1, 27], which could have an influence in the feed intake and negatively affect the fish performance [28]. In order to achieve the minimum requirements, diets with high fishmeal substitution usually need a supplement with synthetic amino acids that increases the price of the diet and could have different adverse effects in nutrient utilisation [29]. Nevertheless, the addition of complementary ingredients such as marine by-products, as opposed or in combination with the amino acid supplementation, seems to be more effective in order to achieve an ideal amino acid profile when alternative vegetable-based diets are used [28]. Indeed, marine by-products, including squid meal or krill meal, are regarded as a high quality protein source, since they show a balanced amino acid profile and contain a considerable amount of free amino acids [28]. Furthermore, these marine ingredients yield several profits, such as acting as feed-attractant that improves feed intake or offsetting some of the deficiencies observed with high plant protein diets for marine carnivorous fish [28, 30, 31].

This work focuses on the impact of a complete replacement of fishmeal during the on-growing period on the intestine of gilthead seabream through the gene expression study of a broad set of genes related to inflammatory response, immune system, gut epithelia integrity, digestive enzymes and peptide transporters. In addition, the effect of the inclusion of marine by-products (squid and krill meal) in seabream plant based diets as a source of marine protein was also assessed in terms of growth parameters and gene expression. The study was supplemented with histological analysis of the foregut, aiming to understand the possible effects in relation to nutrient absorption and inflammatory processes at the morphological level.

## Methods

### Ethics statements

The experimental protocol was reviewed and approved by the Committee of Ethics and Animal Welfare of the Universitat Politècnica de València, following the Spanish Royal Decree 53/2013 and the European Directive 2010/63/UE on the protection of animals used for scientific purposes.

Fish were weighed individually every month during the feed assay, using clove oil with an 87% of eugenol (Guinama ®, Valencia, Spain) as an anaesthetic (1 mg/100 mL of water) to minimize their suffering.

At the end of the growth assay, fish were euthanized by decapitation, after fish were anesthetized with clove oil dissolved in water (1 mg/100 mL of water), thus minimizing their suffering.

### Design of the experiment

#### Rearing system, fish and growth assay

The experiment was conducted at the Universitat Politècnica de València in a recirculating saltwater system (75 m^3^ capacity) with a rotary mechanical filter and a 6 m^3^ capacity gravity biofilter. Nine cylindrical fiberglass tanks with a capacity of 1750 L were used, and water temperature, salinity, dissolved oxygen and pH were as follows: 22.0 ± 0.52 °C, 30 ± 1.7 g/L, 6.5 ± 0.49 mg/L, 7.5–8.5. Water parameters were daily measured. All tanks had similar lighting conditions, with a natural photoperiod (from November to March, average of hours of light: 11 h).

The seabreams were provided by the fish farm PISCIMAR, in Burriana (Castelló, Spain). The feed was given by hand twice a day (at 9:00 and 17:00 h) up to an apparent satiation with a standard commercial (48% crude protein, 23% ether extract, 11% crude ash, 2% crude fibre and 14% nitrogen free extract) diet during the two-month acclimation period to laboratory conditions. The weekly feeding regimen consisted of six days of feeding and one day of fasting. Growth assay started with fish with an average weight of 129 ± 19 g.

Seabream were randomly distributed into 9 fiberglass tanks (twenty fish per tank), and three different experimental diets (a vegetable diet, VM; a fishmeal-based diet, FM and a vegetable diet with marine ingredients, VM+) were randomly assigned to three of them (*n* = 3). Feeding parameters remained the same as during the acclimatation period. The experiment finished when the fish achieved a commercial size, (average weight ~ 350 g), and fish were sacrifice afterwards, 154 days after the beginning of the assay.

Fish weight (g) and survival rate (%) were assessed monthly. Final weight (g) (FW), specific growth rate (% / day) (SGR), feed intake (g/ 100 g fish · day) (FI), feed conversion ratio (FCR), and survival (%) (S) were determined when the experiment was completed. The SGR, the FI and the FCR were obtained taking into account the reported monthly biomass of dead fish.

### Diets

Diets were prepared as pellets by cooking-extrusion with a semi-industrial twin-screw extruder (CLEXTRAL BC-45, Firminy, St Etienne, France); located at Universitat Politècnica de València. The processing conditions were as follows: 0.63 g screw speed, 110 °C and 30–40 atm.

Three isonitrogenous and isoenergetic diets were formulated using commercial ingredients, whose proximal composition was previously analysed according to AOAC (Association of Official Agricultural Chemists) procedures. in the FM diet, the protein was provided by fishmeal, although wheat meal was incorporated as a source of carbohydrates. Synthetic amino acids were not included. The VM diet was based on a mixture of vegetable meals as a protein source and included synthetic amino acids in order to accomplish the minimum requirements of essential amino acids [32]. Finally, VM+ contained a mixture of vegetable meals similar to the VM diet one, but squid meal and krill meal were added to the feed at 10 and 5% level, respectively, reducing the concentration of free amino acid supplementation. These meals were obtained from different companies as by-products: squid meal was provided by Max Nollert (Utrecht, Netherlands) and krill meal by Ludan Renewable Energy (Valencia, Spain).

Amino acids of raw materials and experimental diets were analysed, prior to diet formulation, through a Waters HPLC system (Waters 474, Waters, Milford, MA, USA) consisting of two pumps (Model 515, Waters), an auto sampler (Model 717, Waters), a fluorescence detector (Model 474, Waters) and a temperature control module. Aminobutyric acid was added as an internal standard pattern before hydrolysation. The amino acids were derivatised with AQC (6-aminoquinolyl-N-hydroxysuccinimidyl carbamate). Methionine and cysteine were determined separately as methionine sulphone and cysteic acid after oxidation with performic acid. Amino acids were separated with a C-18 reverse-phase column Waters Acc. Tag (150 mm × 3.9 mm). Proximate composition and essential amino acids of different ingredients are shown in Table 1. The ingredients used, the proximate composition and the essential amino acids of the experimental feeds are included in Table 2.

**Table 1:** Proximal composition and essential amino acid profile of the different aqua feed ingredients

	Fishmeal	Wheat meal	Wheat gluten	Broad Bean meal	Soybean meal	Pea meal	Sunflower meal	Squid meal	Krill meal
Proximate composition (% dry weight)									
Dry matter	90.3	87.8	93.3	89.0	88.1	86.6	89.6	88.0	88.8
Ash	16.8	1.6	0.9	3.0	7.1	3.4	6.7	9.1	10.4
Crude lipid	9.3	1.8	0.9	1.1	2.2	0.8	1.5	15.1	22.5
Crude fiber	0.1	2.8	0.4	9.1	3.6	6.2	18.7	0.9	4.0*
Non-starch polyssaccharides	2.6	23.8	17.2	33.3	32.4	29.9	50.1	4.7	11.0
Crude protein	71.3	11.4	81.0	21.1	49.9	18.7	35.7	71.1	56.1
Essential amino acids (g 100 g^− 1^ dry matter)									
Arginine	5.86	0.38	2.57	1.99	3.66	1.76	3.33	5.90	4.14
Histidine	2.54	0.26	1.45	0.74	1.42	0.58	1.14	1.85	1.26
Isoleucine	3.40	0.36	3.01	1.03	2.33	0.98	1.56	2.28	3.19
Leucine	6.55	0.80	5.79	2.04	4.22	1.78	2.48	4.16	4.67
Lysine	6.01	0.37	1.21	1.92	3.45	1.92	1.39	3.85	3.77
Methionine	2.30	0.22	0.88	0.31	0.92	0.36	1.00	1.76	1.66
Phenylalanine	3.73	0.49	4.31	1.10	2.60	1.11	1.86	2.14	2.97
Threonine	3.55	0.30	1.95	0.94	1.98	0.86	1.52	2.19	2.74
Valine	3.88	0.47	3.26	1.13	2.30	1.06	1.73	2.70	3.12

*4% of chitin

Origin and price of the different ingredients (ingredient, origin, price in € kg ingredient^−1^): FM, Vicens i Batllori S. L. (Girona, Spain), 1.51; WM, Desco S. L. (Museros, Spain), 0.15; WG, Ercros S. A. (Barcelona, Spain), 0.76; BBM, Desco S. L. (Museros, Spain), 0.27; SBM, Desco S. L. (Museros, Spain), 0.31; PM, Desco S. L. (Museros, Spain), 0.23; SFM, Desco S. L. (Museros, Spain), 0.17; SM, Max Nollert (Utrecht, Netherlands), 4.28; KM, Ludan Renewable Energy (Valencia, Spain), 0.25

**Table 2:** Price, ingredients,proximal composition and essential amino acid profile of diets tested in the growth assay

	VM	FM	VM+
Price (€ kg^− 1^)*	0.79	1.09	1.05
Ingredients (g kg^− 1^)			
Fishmeal		589	
Wheat meal		260	
Wheat gluten	295		222
Broad bean meal	41		40
Soybean meal	182		160
Pea meal	41		40
Sunflower meal	158		160
Krill meal			50
Squid meal			100
Fish oil	90	38.1	77.5
Soybean oil	90	92.9	77.5
Soy Lecithin	10	10	10
Vitamin-mineral mix**	10	10	10
Calcium phosphate	38		38
Arginine	5		
Lysine	10		10
Methionine	7		5
Taurine	20		
Threonine	3		
Proximate composition (% dry weight)			
Dry matter	93.9	88.1	92.83
Ash	7.4	10.1	8.8
Crude lipid	19.8	18.5	20
Crude fiber	4.3	0.8	4.6
Non-starch polyssaccharides	21.5	7.7	20.6
Crude protein	45.0	44.2	44.6
Digestible protein***	41.8	42.7	42.0
Essential amino acids (g 100 g^−1^)			
Arginine	3.30	3.39	3.58
Histidine	0.82	1.00	0.81
Isoleucine	1.17	1.47	1.08
Leucine	2.98	3.24	2.45
Lysine	2.26	3.68	2.38
Methionine	1.06	1.16	1.05
Phenylalanine	1.87	1.80	1.76
Threonine	1.44	1.98	1.28
Valine	1.47	2.01	1.32

* The price of the diets was obtained from the individual prices of the different marine and vegetable meals (included in Table 1) and the other ingredients (price in € kg^− 1^ ingredient): soybean oil, 0.63; soy lecithin, 1.15; vitamin-mineral mix, 2.75; calcium phosphate, 2.07; arginine, 7.64; lysine, 1.68; methionine, 3.52; taurine, 2.20; threonine, 1.30

**Vitamin and mineral mix (values are g kg − 1 except those in parenthesis): Premix, 25; choline, 10; DL-a-tocopherol, 5; ascorbic acid, 5; (PO4)2Ca3, 5. The Premix is composed of: retinol acetate, 1,000,000 (IU kg − 1); calcipherol, 500 (IU kg − 1); DL-a-tocopherol, 10; menadione sodium bisulphite, 0.8; thiamine hydrochloride, 2.3; riboflavin, 2.3; pyridoxine hydrochloride, 15; cyanocobalamine, 25; nicotinamide, 15; pantothenic acid, 6; folic acid, 0.65; biotin, 0.07; ascorbic acid, 75; inositol, 15; betaine, 100; polypeptides, 12

***Digestible protein = Crude protein x ADCCP; ADCCP = Apparent Digestibility Coefficient of Protein: ADCCP(VM) = 0,93; ADCCP(FM) = 0,97; ADCCP(VM+) = 0,94

A digestibility experiment was performed after the growth assay, using five randomly selected fish per experimental group and digestibility tanks of 250 l of capacity (one per experimental group). Apparent digestibility coefficient of the crude protein were obtained, according to the Guelph System Protocol [33], by the Chromium Oxide determination method. After two days of fasting, digestibility assay started and lasted 14 days. Fish were fed to satiation once a day (9:00 h) with the same experimental diets containing chromium oxide (50 g kg^− 1^) as an innert marker and uneaten food was then removed from the columns (15:00). Wet faeces were collected from decantation columns just before the next morning feeding and then dried at 60 °C for 48 h prior to analysis. After acid digestion, an atomic absorption spectrometer (Perkin Elmer 3300, Perkin Elmer, Boston, MA, USA) was used for Chromium oxide determination in duplicate in diets and faeces. Apparent digestibility coefficient (ADC) of the crude protein (CP) was calculated as follows (Eq. 1):

1 $$ {ADC}_N\ \left(\%\right)=100\cdotp \left(1-\left(\frac{\% marker\ in\ diet\cdotp \% CP\  in\ faeces}{\% marker\ in\ faeces\cdotp \% CP\  in\ diet}\right)\right) $$

### Economic assessment

The Economic Conversion Rate (ECR) and the Economical Profit Index (EPI) [2] were calculated for each experimental group using Eqs. (2) and (3), respectively. The currency type for economic evaluations was the euro (€). The price of the diets was obtained from the individual prices of the different ingredients. Gilthead seabream sale price was 5.37 € Kg fish^− 1^, based on prices of the Spanish Wholesale market on January 2017. With the aim of showing the impact of the fish mortality on economic profit in on-growing phase, biomass of dead fish was considered and therefore was not included in the total final biomass, and the initial number of fish was used to standarize when the EPI was determined.

2 $$ \mathrm{ECR}\ \left(\text{\EUR} \cdot \mathrm{kg}\ {\mathrm{fish}}^{-1}\right)=\mathrm{FCR}\ \left(\mathrm{kg}\ \mathrm{diet}\cdot \mathrm{kg}\ {\mathrm{fish}}^{-1}\right)\cdot \mathrm{Price}\ \mathrm{of}\ \mathrm{diet}\ \left(\text{\EUR} \cdot \mathrm{kg}\ {\mathrm{diet}}^{-1}\right) $$

3 $$ \mathrm{EPI}\ \left(\text{\EUR} \cdot {\mathrm{fish}}^{-1}\right)=\frac{\mathrm{Final}\ \mathrm{biomass}\left(\mathrm{kg}\ \mathrm{fish}\right)\cdot \mathrm{Sale}\ \mathrm{price}\ \left(\text{\EUR} \cdot \mathrm{kg}\ {\mathrm{fish}}^{-1}\right)-\Delta \mathrm{biomass}\ \left(\mathrm{kg}\ \mathrm{fish}\right)\cdot \mathrm{ECR}\ \left(\text{\EUR} \cdot \mathrm{kg}\ {\mathrm{fish}}^{-1}\right)}{\mathrm{Initial}\ \mathrm{number}\ \mathrm{of}\ \mathrm{fish}} $$

### Sampling

In order to assess gene expression and histological changes throughout the intestinal tract, intestinal samples from three fish per tank were sampled at the end of the growth assay after one day of fasting (40 h after the last feed). Based on the separation on sections proposed in previous researches [34], three different sections were considered but only pieces of foregut (FG) and hindgut (HG) were collected and stored in RNA later(Ambion Inc., Huntingdon, UK) at 4 °C overnight and then at − 20 °C until RNA extraction. Pieces of FG section (two fish per tank, *n* = 6) were stored in phosphate buffered formalin (4%, pH 7.4) for the histological assessment.

### Gene expression

#### RNA extraction and cDNA step

Total RNA was extracted from FG and HG tissues by traditional phenol/chloroform extraction, using TRIzol Reagent (Invitrogen, Spain), and then purified and treated with DNase I using NucleoSpin® RNA Clean-up XS kit (Macherey-Nagel, Düren, Germany), according to guide instructions. Total RNA concentration, quality and integrity were evaluated using a NanoDrop 2000C Spectrophotometer (Fisher Scientific SL, Spain) and samples were stored at − 80 °C until complementary DNA (cDNA) synthesis.

cDNAwas synthetized from 1 μg of total RNA input using the qScript cDNA Synthesis Kit (Quanta BioScience), according to the manufacturer’s instructions, using the Applied Biosystems 2720 Thermal Cycler. The cycling conditions were 22 °C for 5 min, 42 °C for 30 min, and 85 °C for 5 min. Total RNA samples were stored at − 80 °C until gene expression was analysed.

### Measurement of gene expression by SYBR green assay real time quantitative RT-PCR (qPCR)

#### Reference and target genes

Four candidate reference genes (ef1α, gapdh, rps18, βact; Table 3) were tested to be used as housekeeping genes in the gene expression assay. The stability of these genes was determined using six cDNA pooled samples, obtained each one from combine equally volumes of cDNA samples from the same section in a given experimental group. Ribosomal protein s18 (*rps18*) and β-actin (*βact*) were selected as reference genes for the normalization of gene expression based on the stability of its expression in the cDNA pools and the cDNA specificity in the amplification, confirmed by melting curve analysis [see Additional file 1].

**Table 3:** Primer sequences of candidate genes (reference and target genes) in the RT-qPCR assay

Gene	Abbreviation	GeneBank ID	Primer Forward	Primer Reverse	Lenght	Reference
REFERENCE GENES						
Elongation Factor 1α	*ef1α*	AF184170	CTGTCAAGGAAATCCGTCGT	TGACCTGAGCGTTGAAGTTG	87	[16, 20]
Glyceraldehide 3-phosphate dehydrogenase	*gapdh*	DQ641630	CCAACGTGTCAGTGGTTGAC	AGCCTTGACGACCTTCTTGA	80	[17]
Ribosomal Protein S18	*rps18*	AM490061	AGGGTGTTGGCAGACGTTAC	CGCTCAACCTCCTCATCAGT	97	[17]
β-Actin	*βact*	X89920	TCTGTCTGGATCGGAGGCTC	AAGCATTTGCGGTGGACG	113	[19]
TARGET GENES						
Interleukin 1β	*il1β*	AJ277166	GCGACCTACCTGCCACCTACACC	TCGTCCACCGCCTCCAGATGC	131	[17]
Interleukin 6	*il6*	AM749958	AGGCAGGAGTTTGAAGCTGA	ATGCTGAAGTTGGTGGAAGG	101	[16]
Interleukin 8	*il8*	JX976619	GCCACTCTGAAGAGGACAGG	TTTGGTTGTCTTTGGTCGAA	164	[16, 20]
Tumor Necrosis Factor α	*tnfα*	AJ413189	CAGGCGTCGTTCAGAGTCTC	GAGATCCTGTGGCTGAGAGG	83	[17]
Cyclooxygenase 2	*cox2*	AM296029	GAGTACTGGAAGCCGAGCAC	GATATCACTGCCGCCTGAGT	192	[16, 20]
Caspase 1	*casp1*	AM490060	ACGAGGTGGTGAAACACACA	GTCCGTCTCTTCGAGTTTCG	92	[16]
Intestinal Mucin	*imuc*	JQ277712	GTGTGACCTCTTCCGTTA	GCAATGACAGCAATGACA	102	[19]
Mucin 2	*muc2*	JQ277710	ACGCTTCAGCAATCGCACCAT	CCACAACCACACTCCTCCACAT	90	[19]
Mucin 2-like	*muc2l*	JQ277711	GTGTGTGGCTGTGTTCCTTGCTTTGT	GCGAACCAGTCTGGCTTGGACATCA	67	[19]
Mucin 13	*muc13*	JQ277713	TTCAAACCCGTGTGGTCCAG	GCACAAGCAGACATAGTTCGGATAT	67	[19]
Mucin 19	*muc19*	JQ277715	TGCTTGCTGATGACACAT	TTCACATAGGTCCAGATATTGA	128	[19]
Immunoglobulin M	*igm*	JQ811851	TCAGCGTCCTTCAGTGTTTATGATGCC	CAGCGTCGTCGTCAACAAGCCAAGC	131	[18]
Occludin	*ocl*	JK692876	GTGCGCTCAGTACCAGCAG	TGAGGCTCCACCACACAGTA	81	[16, 20]
Tubulin	*tub*	AY326430	AAGATGTGAACTCCGCCATC	CTGGTAGTTGATGCCCACCT	98	[16]
α-Amylase	*αamy*	AF316854	TGGTGGGACAATCAGAGTCA	GTCCAGGTTCCAGTCGTCAT	85	[16, 20]
Alkaline phosphatase	*alp*	AY266359	TTACTGGGCCTGTTTGAACC	GATCTTGATGGCCACTTCCAC	102	[16, 20]
Trypsin	*tryp*	AF316852	GGTCTGCATCTTCACCGACT	AAAGGCAGCAGAGTGATGGT	85	[20]
Peptide transporter 1	*pept1*	GU733710	TTGAACATAACGTCGGGTGA	AATTTTGCATTTCCCTGTGG	92	[16]

Expression stability of reference genes in individualized samples was determined using the BestKeeper program [35], which reports a standard deviation (SD[±Cq]) lower than 1 for both genes (0.54 for *rps18* and 0.68 for *βact*, *p* < 0.05) and Cq arithmetic means of 20.19 ± 1.46 and 17.96 ± 1.6 for *rps18* and *βact*, respectively. The BestKeeper’s calculated variations in the reference genes are based on the arithmetic mean of the Cq values.

Eighteen candidate target genes (Table 3) were previously tested by RT-qPCR. The proinflammatory cytokines genes *il1β*, *il6* and *il8*, and other proinflammatory molecules, as *tnfα*, *casp1* AND *cox2* were included due to their relevance as inflammation markers [16, 20]. Genes encoding different mucins (*imuc*, *muc2*, *muc2L*, *muc13* and *muc19*), which contribute to protect the intestine epithelium against a broad spectrum of damages [19], and specific antibodies (*igm*) were also chosen to assess the response of the innate and adaptive immunity, respectively. A tight junction protein, such as *ocl*, and an essential component of microtubules such as *tub* [16] were included in the expression pretesting due to their involvement in the maintenance of the epithelial gut integrity. Regarding the selected genes encoding digestive enzymes and nutrient transporters, *αamy* and *tryp* are digestive enzymes responsible for hydrolysis of carbohydrates and proteins, respectively, and *pept1* is a peptide transporter at the brush border membrane of the enterocytes with an important role in the intestinal absorption [36]. Finally, the gene expression of the *alp*, responsible of removing the phosphate groups of many different molecules [37], was also determined.

This preliminary gene expression test was performed using the cDNA pooled samples used in the reference gene evaluation [see Additional file 2]. Target genes for the further individualized assesment were selected based on their function, potential fold-change differences between diets and intestine segments (significant differences cannot be determined by an statistical analysis since *n* = 1), gene expression level and nonspecific amplifications. Later on, relative gene expression of the nine selected genes (*il1β*, *il6*, *cox2*, *igm*, *imuc*, *ocl*, *pept1*, *tryp*, *alp*) was determined at the FG and at the HG in nine fish per dietary treatment.

### RT-PCR assay conditions

All qPCR assays and expression analyses were performed using the Applied Biosystems 7500 Real-Time PCR with SYBR® Green PCR Master Mix (ThermoFisher Scientific, Waltham, Massachusetts, USA). The total volume for every PCR reaction was 10 μL, performed from diluted (1:50) cDNA template (1 μL), forward and reverse primers (10 μM, 1 μL), SYBR® Green PCR Master Mix (5 μL) and nuclease-free water up to 10 μL.

After an initial Taq activation of polymerase at 95 °C for 10 min, 42 cycles of PCR were performed with the following cycling conditions: 95 °C for 10 s and 60 °C for 20 s in all genes, except for *alp* (with annealing and extension step at 55 °C). In order to evaluate assay specificity, a melting curve analysis was directly performed after PCR by slowly increasing the temperature (1 °C / min) from 60 to 95 °C, with a continuous registration of changes in fluorescent emission intensity.

The analysis of the results was carried out using the 2^-ΔΔCt^ method [24]. The target gene expression quantification was expressed relative to the expression of the two reference genes (*rps18* and *βact*). A cDNA pool from all the samples was included in each run and acted as a calibrator, and a non-template control for each primer pair, in which cDNA was replaced by water, was run on all plates. Reference and target genes in all samples were run in duplicate PCR reactions.

### Histological analysis

Fragments of FG fixed in formalin were routinely dehydrated in ethanol, equilibrated in UltraClear (Bio-Optica Milano s. p. a., Milan, Italy), and embedded in paraffin according to standard histological techniques. Transverse sections were cut with a thickness of 5 μm with a microtome Shandom Hypercut (four sections per paraffin block were obtained) and dyed with the haematoxylin-eosine staining method. A total of 72 FG sections, obtained from 18 different paraffin blocks (*n* = 6), were analysed under the light microscope (Nikon, Phase Contrast Dry JAPAN 0.90), focusing on possible inflammatory changes and other disorders.

**Fig. 1:**
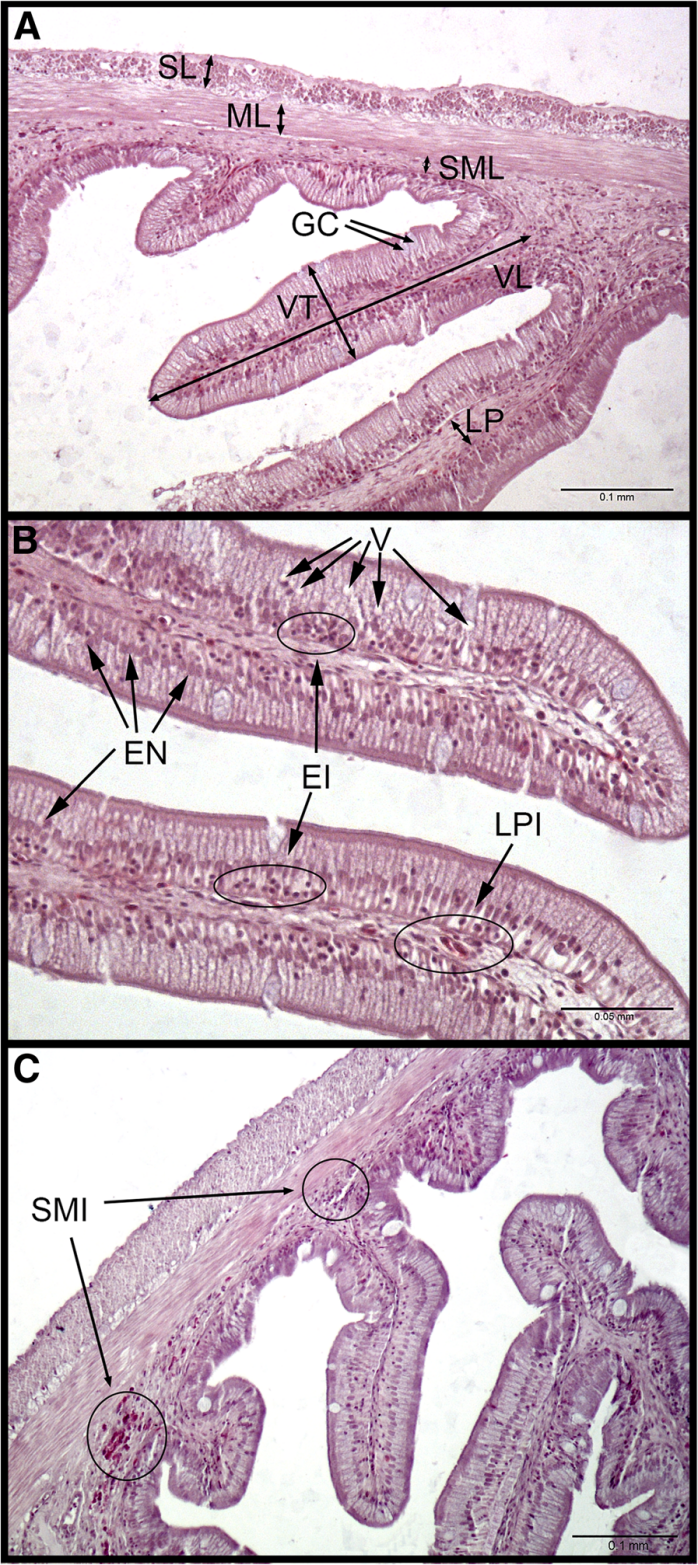
Evaluation and scoring system used to assess histological parameters of gilthead seabream foregut. **a** Measurements performed in a foregut histological section (20×). **b** Detail of villi with a certain grade of infiltration of the lamina propia and the epithelia. Enterocytes nuclei were displaced in some cases. Epithelial vacuolization can also be observed in a normal grade (40×). **c** Enterocytes showed aligned nuclei in a basal position. Villi presented a low grade of infiltration of their lamina propia and of the epithelia, and low vacuolization. A certain grade of infiltration in the submucosa layer can be observed (20×). SL, ML, SML, VL, VT and number of GC were measured six times per section, and averages were obtained for each section (six sections per group, *n* = 6). V, EN, EI, LPI and SMI were assessed in each section (n = 6) using the following scoring system: V, normal (1) to hypervacuolated (4); EN, basal (1) to apical (4); EI, low (1) to markedly increased (4); LPI, low (1) to markedly increased (4); SMI, low (1) to markedly increased (4). SL, serous layer; ML, muscular layer; SML, submucosa layer; VL, villi length; VT, villi thickness; LP, lamina propria; GC, goblet cells; V, supranuclear absorptive vacuoles; EN, enterocytes nuclei; EI, epithelial infiltration; LPI, lamina propria infiltration; SMI, submucosa infiltration

A combination of different criteria reported by several authors [7, 9, 38–40] was used to measure the following parameters at FG sections: serous layer (SL), muscular layer (ML), submucosa layer (SML), villi length (VL), villi thickness (VT) and lamina propria thickness (LP), and number of goblet cells per villus (GC). Six measurements per section in each parameter were performed and average means were obtained for each sample (*n* = 6). Moreover, a continuous scoring system (Fig. 1), ranging from 1 to 4, was used to assess the supranuclear vacuolization on the epithelia (V), the position of the nuclei of the enterocytes (EN) and the lymphocytic infiltration of the epithelial layer (EI), the lamina propria (LPI) and the submucosa (SMI) in each sample (n = 6).

### Statistics

Statistical data analyses were carried out with Statgraphics © Centurion XVI software (Statistical Graphics Corp., Rockville, MO, USA).

Differences in fish weight and survival between dietary groups were monthly evaluated by simple analysis of variance, considering the tank as the experimental unit. At the end of the growth trial, economic indeces (ECR and EPI) and livestock data (FW, SGR, FCR, FI and S) were subjected to simple variance analysis. Each group in the calculation represented the combined group of fish per single tank (triplicate tanks per treatment). Student Newman-Keuls test was used to assess specific differences among dietary groups at the 0.05 significance level. Descriptive statistics are shown as the mean ± pooled standard error of the mean (SEM).

Relative gene expression data was statistically analysed by two-way analysis of variance using Newman-Keuls test. Differences in expression were considered statistically significant when *p* < 0.05. Data was expressed with the mean and the standard error for each gut section and experimental group. Differences in the gene expression between sections within each group, between experimental groups, and between same sections in different dietary groups were determined.

Finally, histological measurements in foregut were showed as the mean ± standard error of the mean and it was analysed through an analysis of variance (ANOVA), with a Newman-Keuls test for the comparison of the means and a level of significance set at *p* < 0.05. Principal Component Analysis was used to analyse the histological scored parameters of gut (V, EN, EI, LPI and SMI). Statistical differences between experimental groups were estimated by ANOVA using the first and second Principal Components of the Principal Component Analysis, with a Newman-Keuls test (*p* < 0.05).

## Results

### Economic indices

Statistically differences were determined in the ECR between the groups FM and VM (*p* = 0.0473), whilst the EPI was greater in the groups FM and VM+ (*p* = 0.0167) (Table 4). Differences in the ECR can be explained by the greater cost of the FM feed, while the lower number of fish at the end of the growth assay in the different tanks assigned to the VM treatment led to a lower EPI (*p* = 0.0167) in this dietary group.

**Table 4:** Growth and economic indices of seabream fed experimental diets at the end of the experiment

	FM	VM	VM+	SEM	*p*-value
Initial Weight (g)	131.2	127.2	129.6	4.1	0.9023
Final Weight (g)	393.1	360.4	384.6	13.2	0.2703
Specific Growth Rate (% / day)	0.72	0.69	0.73	0.03	0.6562
Feed Intake (g / 100 g fish · day)	1.35	1.38	1.33	0.02	0.2758
Feed Conversion Ratio	2.14	2.40	2.08	0.10	0.1536
Survival (%)	88.3^a^	60.0^b^	86.7^a^	5.18	0.0141
Economic Conversion Rate (€ / kg fish)*	2.35^a^	1.90^b^	2.24^ab^	0.10	0.0473
Economic Profit Index (€ / fish)*	1.36^a^	0.99^b^	1.33^a^	0.07	0.0167

*Price of diets: VM = 0.79 €; FM = 1.09 €; VM+ = 1.05 €. Sale price of gilthead seabream = 5.37 € kg fish^− 1^

Means of triplicate groups (*n* = 3). Data in the same row with different superscript letters differ at *p* < 0.05

Initial Weight (g); Final Weight (g); Specific Growth Rate (% day^− 1^) = 100 •·ln (final weight (g)/ initial weight (g)) / days; Feed Intake (g 100 g fish^− 1^ day^− 1^) = 100 • feed consumption (g) / (average biomass (g)· days); Feed Conversion Ratio = feed offered (g) / weight gain (g); Survival (%) = 100 • (final number of fish / initial number of fish); Economic Conversion Rate (€ kg fish^− 1^); Economical Profit Index (€)

*SEM* Standard error of the mean

### Growth assay and growth indices

**Fig. 2:**
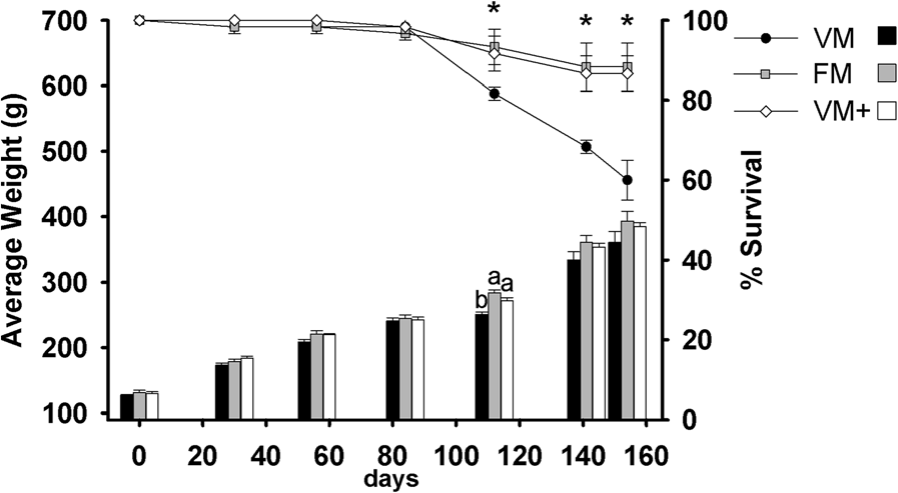
Average weight (g) and survival rate (%) evolution of gilthead seabream along the assay period. Average weight mean and standard error (bars) and survival rate (line) of each experimental group were displayed in different colours (Black: VM; Grey: FM; White: VM+). Different superscripts on the bars indicate significant statistical differences in the average weight during the growth trial (*p* < 0.05). Data are means of triplicate groups (*n* = 20). Asterisks indicate the existence of significant differences in the survival rate along the assay at *p* < 0.05

Differences were observed in the average weight of fish after 112 days from the beginning of the growth assay (*p* = 0.0042), registering greater weight in those fish fed FM and VM+ than in fish fed VM (Fig. 2), although no significant differences were observed in subsequent sampling points and at the end of the feeding trial (Table 4). Survival rate of fish fed VM began to decrease after 112 days (*p* = 0.0332) of the experiment in comparison to the rates observed in the other two groups (FM and VM+). Survival rate continued decreasing at VM group as the growth trial progressed, but no disease signs were reported in dead fish. No differences were observed in the growth parameters, which are shown in Table 4.

### Gene expression

#### Inflammation and immune system genes

**Fig. 3:**
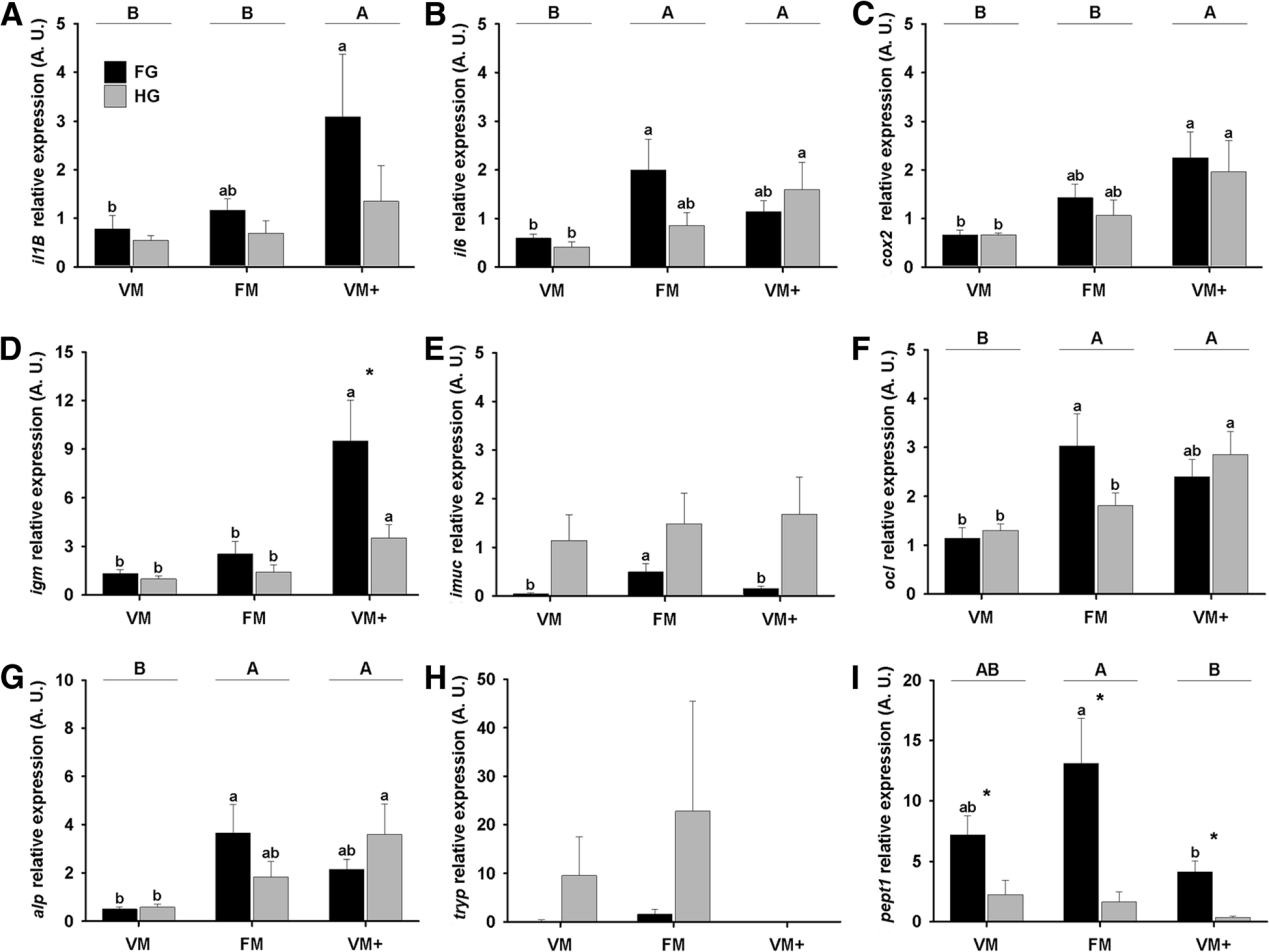
Relative gene expression in the intestine of gilthead seabream fed different experimental diets. **a** Interleukine-1β (*il1β*); **b** Interleukine-6 (*il6*); **c** Cyclooxigenase-2 (*cox2*); **d** Intestinal Mucin (*imuc*); **e** Immunoglobulin M (*igm*). **f** Occludin (*ocl*); **g** Alkaline Phosphatase (*alp*); **h** Trypsin (*tryp*); **i** Peptide Transporter 1 (*pept1*). Bars represent relative gene expression (mean + standard error, *n* = 9), for each group, in the foregut (FG, black bars) and the hindgut (HG, grey bars). Superscript letters on the bars indicate differences between experimental groups in each section, at *p* < 0.05. Asterisks indicate differences between intestinal sections in each experimental group, at *p* < 0.05. Capital letters at the top of the graph indicate differences between experimental groups, regardless the intestinal section (*n* = 18, *p* < 0.05), when interaction between factors (diet and section) is not significative (Table 5)

The diet was determined as a significant factor affecting the expression of *il1β*, *il6*, *cox2* and *igm* (Table 5). Fish fed VM and FM reported lower expression levels of *il1β* (Fig. 3a), *cox2* (Fig. 3c) and IgM (Fig. 3d) in comparison to VM+ group, and a lower expression of *il6* (Fig. 3b) was observed in the VM group. IgM and *i-muc* relative expression were affected by the section (Table 5): *igm* (Fig. 3d) had a higher expression in FG than in HG, specially in the group VM+, and *i-muc* (Fig. 3e) reported a remarkably higher expression in the HG.

**Table 5:** *p*-values* determined for diet, intestinal section and the interaction between both factors on the gene expression assay

Gene	Diet	Section	Diet x section
*il1β*	< 0.05	ns	ns
*il6*	< 0.05	ns	ns
*cox2*	< 0.01	ns	ns
*igm*	< 0.0001	< 0.05	< 0.05
*imuc*	n.s.	< 0.01	ns
*ocl*	< 0.01	ns	ns
*alp*	< 0.01	ns	ns
*tryp*	ns	ns	ns
*pept1*	< 0.05	< 0.0001	ns

*p-values were obtained using the Student Newman-Keuls test in a two way analysis of variance

*ns* Non significant

### Structural, enzyme and nutrient transport genes

Expression of *ocl*, *alp* and *pept1* was influenced by the diet (Table 5). The VM group showed a lower expression of *ocl* (Fig. 3f) and alp (Fig. 3g) in comparison to the other two groups. Additionally, this group showed a lower expression of pept1 (Fig. 3i) in comparison to FM, but a greater expression compared to VM+.The relative gene expression of *tryp* (Fig. 3h) showed a large individual variation and no differences were found at diet (*p* = 0.4677) or section level (*p* = 0.2036). Finally, the expression of *pept1* was also affected by the section (Table 5), being overexpressed in the FG compared to HG in all experimental groups (Fig. 3i).

### Histological analysis

Fish fed VM exhibited thinner villi and lamina propria than the fish fed the FM diet (Table 6). No differences were determined in the thickness of the three layers of the intestinal wall, nor in the length of the villi and the thickness of the lamina propria. The number of GC was increased in many of the fish fed the vegetable diets, especially for the fish fed VM+, although no significant differences were determined between dietary groups.

**Table 6:** Dietary effect on the histomorphology of the foregut of gilthead seabream

	FM	VM	VM+	SEM	p-values
Serous layer (μm)	50.3	52.9	61.7	5.2	0.1275
Muscular layer (μm)	58.4	56.6	55.8	5.4	0.8000
Submucosa layer (μm)	44.3	40.4	43.0	4.5	0.6689
Villi length (μm)	621.7	512.2	568.7	57.4	0.2545
Villi thickness (μm)	101.1^a^	85.2^b^	93.4^ab^	4.1	0.0009
Lamina propria (μm)	14.2^a^	9.9^b^	14.0^a^	1.3	0.0001
Goblet cells	3.2	4.4	4.9	1.1	0.1222

Means were calculated from the average mean of each sample (n = 6). Data in the same row with different superscript letters differ at *p* < 0.05

*SEM* Standard error of the mean

**Fig. 4:**
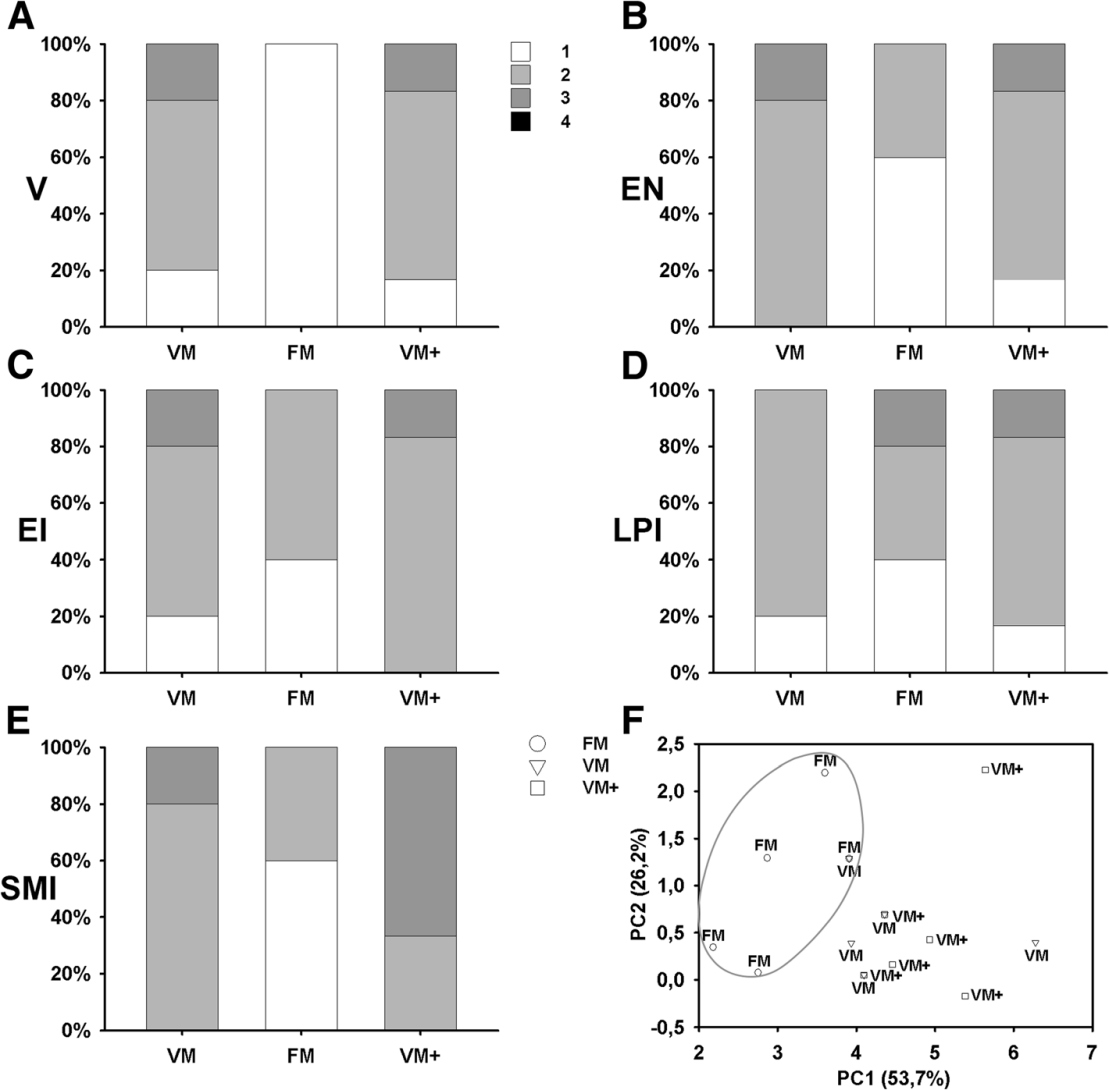
Histological assessment of foregut sections of gilthead seabream fed different experimental diets, according to Fig. 1**.** Frequency bar charts showing differences in **a** supranuclear absorptive vacuolization (V), **b** enterocytes nuclei (EN), **c** enterocytes infiltration (EI), **d** lamina propria infiltration (LPI) and **e** submucosa infiltration (SMI). **f** Dispersion graph representing values of the first and second components for each foregut section assessed, obtained from the Principal component analysis of histological foregut scores according to diet. Only sections evaluated in all parameters were included in the Principal component analysis (*n* = 5 for VM and FM, n = 6 for VM+)

Assessment by scoring of different parameters of the gut (Fig. 4) revealed differences on the number of supranuclear absorptive vacuoles in the epithelial layer (V), the displacement of the enterocytes nuclei to apical positions (EN), and the degree of inflammatory cells infiltration in the submucosa layer (SMI). In these three assessed parameters, related with the inflammatory status,higher values were reported in the foregut sections belonging to VM and VM+ groups. Dispersion graph (Fig. 4) based on the First and Second component values obtained from the Principal Component Analysis, showed evident differences among the sections belonging to FM group and the sections from groups fed with plant-based diets. First Component of the Principal Component Analysis explained the 53,7% of the variability and was related with the degree of inflammation. In this sense, an ANOVA taking this First Component as a variable confirmed the existence of significant differences (*p* = 0.0063) between FM sections and sections of the groups of fish fed vegetable diets (VM and VM+).

Summarizing, the VM group registered greater mortality and lower expression of *il6*, *ocl*, *alp* and *pepT1* at intestinal level, while the VM+ group registered higher expression of *il1β*, *cox2* and *igm* and lower expression of *pept1*. At histological level, both dietary groups (VM and VM+) reported thinner villi in the foregut compared to the FM group, an apical displacement of the enterocytes nuclei and higher vacuolization and cellular infiltration in the submucosa.

## Discussion

### Zootecnical and economical parameters

Based on the evolution of mean weight and survival rates, the impact of the different feeds on the growth and survival can be observed from 112 days of the growth assay. However, although survival rates of fish fed the VM diet decreased from this time until the end of the trial, no significant differences in terms of mean weight were registered at the 140 and 154 days. Dead fish found in the VM tanks in the final stage of the assay were mainly the smallest fish in these tanks, which could explain the disappearance of significant differences in the mean weight at the end of the trial. Variability in the different experimental groups prevents differences in growth indices,, specially on the FCR. The less growth performance and greater mortality reported in the VM group are manifested in the economic indices. In this sense, FM and VM+ diets showed a similar efficiency under a economical point of view.

### Intestinal status

Fish perfomance, including growth and survival, could be compromised by alterations in the intestinal homeostasis [11].

Fishmeal replacement by different vegetable sources has been associated with occurrence of gut inflammation in different species [41, 42]. Previous research have reported the up-regulation of the expression of different inflammatory markers [22, 23, 43, 44], higher grade of cell infiltration in the submucosa and changes in the expression of genes related with several processes, including antioxidant defences, cell differentiation, epithelial permeability, immunity and mucus production [22, 43] in response to moderate and high levels of plant protein sources inclusion.

In the present work, the group VM+, in which fishmeal was totally replaced by plant sources and squid and krill meal were included at 15% level, reported the up-regulation of pro-inflammatory markers (il1*β* and *cox2*) and *igm* compared to the FM group. The increase of gene expression in relation to inflammatory mediators has been linked to the regulation of the inflammation [20] and the activation of the innate immunity in response to infection [45], and it has been observed as a common response against low fishmeal based diets in several species [23]. Although IgT has been recently suggested as the main inmunoglobulin in the mucosal responses in gilthead seabream [46], IgM plays a key role in the gut mucosal immune reactions against pathogens or environmental stress, and also in the triggering of the humoral response [18, 47]. Additionally, high levels of IgM in the gut mucosa of fish fed with plant sources based diets have been reported [48]. Thus, the up-regulation of these genes could reflect that fish fed VM+ were developing an inflammatory process at the intestinal mucosa level, and are able to maintain an active local immune system after the growth trial.

In contrast, this up-regulation is not observed in the VM group, which showed a lower expression of different pro-inflammatory markers and other genes related with the immune defence (*igm, alp*) and the regulation of epithelial permeability (*ocl*), even lower compared to the FM group. Occludin has been suggested as a key protein in the epithelial integrity maintenance and in the regulation of permeability and other properties of the epithelial barrier [49], being a marker of integrity of the tight junction between the enterocytes, and its underexpression could suggest deficiencies in the regulation of the gut inflammatory response [16, 20]. Importance and physiologic function of alkaline phosphatase in digestion and a possible dietary regulation of its expression remain unclear, but it has been described as a gut mucosal defense factor, which seems to be implicated in the mucosal defence through the dephosphorylation of the lipopolysaccharides from the endotoxins of gram-negative bacteria [50]**.** Microbial lipopolysaccharides upregulates *alp* and its activity reduces toxicity of lipopolysaccharides [37], preventing from excessive inflammation in response to commensal microbes and helping to maintain the balance and integrity of the intestinal epithelial barrier [51].

The down-regulation of the expression of the genes could reflect that fish fed the VM diet were not triggering an inflammatory response at the end of the growth trial, as well as certain grade of immune mechanism suppression at local level, maybe evidencing an stress response. This depressed status could explain the higher mortality reported in this group and it could be linked with microbial imbalances that have been described in response to total fishmeal replacement in gilthead seabream [12].

In this sense, inclusion of great amounts of plant protein sources in aqua feeds for carnivorous species can be considered as a chronic stress factor, triggering a reponse by the host [52], which redirects more energy and resources to face with the stressor [53]. After long periods, immune mechanisms and other pathways that demand a continuous energy supply can be affected, leading to depressive or suppresive effects [52], leading to a chronic stress status. The suppresion of inflammatory and immune mechanisms in response to long term feeding high plant protein diets has been observed in previous research in different species [54, 55], including the gilthead seabream [9], and a differential response was also observed in different intestinal sections [22]..

Exposition to antinutrients included into the vegetable-based diets (VM+, and specially, in VM) throughout the growth assay could initially determine a prolongued inflammatory reaction in both experimental groups, demanding an additional energy expenditure that fish fed VM are not able tot sustain. Therefore, differences in the inflammatory and immune status of the gut between the VM and VM+ group at the end of the growth assay might be explained by dietary composition.The VM diet only includes vegetable meals, and synthetic amino acids were added in order to comply minimum amino acid requirements [32], while in the VM+ diet squid and krill meal —which have higher quality protein than vegetable meals and could improve essential amino acid profile in terms of bioavailability— were included at 10% and 5% levels, respectively, and the amount of synthetic amino acids was lower. This inclusion of marine by-products at 15% level could favour the maintenance of an active gut proinflammatory response along the experiment, while the VM diet could be a deficient diet from a nutritional point of view and fish could be unable to meet the energy requirements to sustain the inflammatory response during all the growth trial. Chitin, which is present in the krill meal at 4%, could increase the activity of the seabream immune system [56]. Composition in fiber, non-starch polysaccharides and fatty acids was very similar in both experimental diets and did not seem to be the reason of the observed differences.

The higher expression of *pept1* at the FG of fish confirms that this is the main production site and the intestinal section in which most of the absorption of small peptides takes place in gilthead seabream [36]. The downregulation of the peptide transporter in the anterior intestine of fish fed VM, and especially of fish fed VM+, could be related to a greater presence of non-starch polysaccharides, saponins or other antinutrients in the vegetable based diets, which could alter the gut integrity and reduce the gastrointestinal passage of the food [57], and also to a lower digestibility of vegetable protein, which possibly contributes to a lower small peptide transport.

Finally, some possible minor inflammatory signs were observed at histological level in the present work in fish fed with both plant protein based diets (VM and VM+), which could suggest that fish fed VM could develop an inflammatory reaction at certain point of the growth assay, before a possible suppression of inflammatory and immune mechanisms. Modifications include a higher grade of vacuolization in the epithelia and an increase of cell infiltration in the submucosa layer. Presence of supranuclear absorptive vacuoles in the epithelial layer is normal, but their excessive accumulation could be related to changes in the function of enterocytes [58], and it is often accompanied with evident signs of inflammation, as immune cell infiltration, as it has been observed in previous studies in response to different experimental diets in different species and in different segments of the gut [4, 7, 59–62]. Moreover, villi with a great number of GC were observed in the gut of fish fed diets containing vegetable meals, especially on the VM+ group, which were not observed in the foregut of fish from the FM group., However, no statistical differences were determined, because villi with a reduced number of GC were observed in all experimental groups.. The increase in the number of GC has been noticed in rainbow trout [59], likewise in seabream fed with vegetable-based diet [6, 11], suggesting a possible alteration of secretory processes. GC secreted a mucus gel that covered the epithelium of the intestinal tract [63], so that the thicker mucus layer observed in fish fed vegetable based diets during the sampling process is consistent with these findings, although no differences were reported between experimental groups in the *imuc* expression in the HG, were it is constitutively expressed accordin to previous research [19]. However, no enteritis features in the FG were found, which is in accordance to previous studies [7]. In this sense, tolerance to antinutrients, which may be the cause of enteritis [59], seems to depend on species [4], and gilthead seabream seems to tolerate high levels of plant sources in diets without intestinal structural damage [9, 64], and only moderate changes, without pathological signs, have been observed in most research works [4, 7, 9, 25, 43, 44, 64]. In this sense, a higher degree of cellularity and the widening of the lamina propria -described as signs of inflammation- of fish fed vegetable diets, were not noticed in the present experiment, but similar observations were also made [6, 10, 11] in feeding trials with high levels of fishmeal replacement, so this point must be clarified.

Finally, thinner villi observed in the FG of fish fed with VM can affect the nutrients absorption capacity, although impact on growth may be more related with the allocation of energy to face with an prolongued inflammatory status than with histomoprhological changes. However, a similar effect with great amounts of fishmeal replacement by plant sources has been observed [10] and further investigation should be also performed on this issue to explain that response.

## Conclusion

Total replacement of fishmeal by vegetable protein sources in diets for the on-growing of gilthead seabream had a negative impact on long-term fish survival under the experimental conditions, maybe caused by a lack of gut mucosal immune response derived from a lingering poor nutritional status. The inclusion of squid and krill meal in vegetable-based diets seemed to produce a long-term inflammation response in the gut, but no negative effects on fish survival were reported. However, development of vegetable-based diets that do not cause gut inflammatory reactions is needed in order to ensure, not only growth and survival, but also health status and welfare of fish.

## Additional files

Additional file 1: Cq values reported in cDNA pooled samples when evaluating candidate reference genes. It Includes Cq determined for different candidate reference genes in six different cDNA pooled samples, and the average and standard desviation. (XLSX 9 kb)

Additional file 2: Relative gene expression of candidate target genes in cDNA pooled samples. A) Interleukine-1β (*il1β*), Interleukin-6 (*il6*) and Interleukine-8 (*il8*); B) Tumor Necrosis Factor–α (*tnfα*), Caspase 1 (*casp1*), Cyclooxigenase-2 (*cox2*); C) Mucin 2 (*muc2*), Mucin 2-like (*muc2L*), Mucin 13 (*muc13*); D) Intestinal Mucin (*imuc*), Mucin 18 (*muc18*), Immunoglobulin M (*igm*); E) Occludin (*ocl*) and Tubuline (*tub*); F) α-Amylase (*αamy*) and Alkaline Phosphatase (*alp*); G) Trypsin (*tryp*) and Peptide Transporter 1 (*pept1*). Different genes are represented with different colours. Bars represent relative gene expression of cDNA pools (one per section and treatment), in the foregut (FG) and the hindgut (HG). cDNA pool of the foregut of fish fed VM was used as a calibrator. (TIF 169 kb)

## Abbreviations

*alp*: Alkaline Phosphatase
ANOVA: Analysis of variance
*casp1*: Caspase 1
*cox2*: Cyclooxygenase 2
CP: Crude protein
ECR: Economic Conversion Rate
*ef1α*: Elongation Factor 1α
EI: Lymphocytic infiltration of the epithelilal layer
EPI: Economical Profit Index
FCR: Feed conversion ratio
FG: Foregut
FI: Feed intake
FM: Fishmeal-based diet
FW: Final weight
*gapdh*: Glyceraldehide 3-phosphate dehydrogenase
GC: Goblet cells
HG: Hindgut
*igm*: Immunoglobulin M
*il1β*: Interleukin 1β
*il6*: Interleukin 6
*il8*: Interleukin 8
*imuc*: Intestinal Mucin
LP: Lamina propria thickness
LPI: Lymphocytic infiltration of the lamina propria
ML: Muscular layer
*muc13*: Mucin 13
*muc19*: Mucin 19
*muc2*: Mucin 2
*muc2L*: Mucin 2-like
N: Position of the nuclei of the enterocytes
*ocl*: Occludin
*pept1*: Peptide Transporter 1
*rps18*: Ribosomal protein S18
S: Survival
SEM: Standard error of the mean
SGR: Specific growth rate
SL: Serous layer
SMI: Lymphocytic infiltration of the submucosa
SML: Submucosa layer
*tnfα*: Tumor Necrosis Factor α
*tryp*: Trypsin
*tub*: Tubulin
V: Supranuclear vacuolization
VL: Villi length
VM +: Vegetable diet with marine ingredients
VM: Vegetable diet
VT: Villi thickness
*αamy*: α-Amylase
*βact*: β-Actin
