# Supplementary figures and images for: Long-term feeding with high plant protein based diets in gilthead seabream (Sparus aurata, L.) leads to changes in the inflammatory and immune related gene expression at intestinal level
Source: BMC Vet Res. 2018 Oct 3;14:302. doi: 10.1186/s12917-018-1626-6 (PMC6171182; doi:10.1186/s12917-018-1626-6)

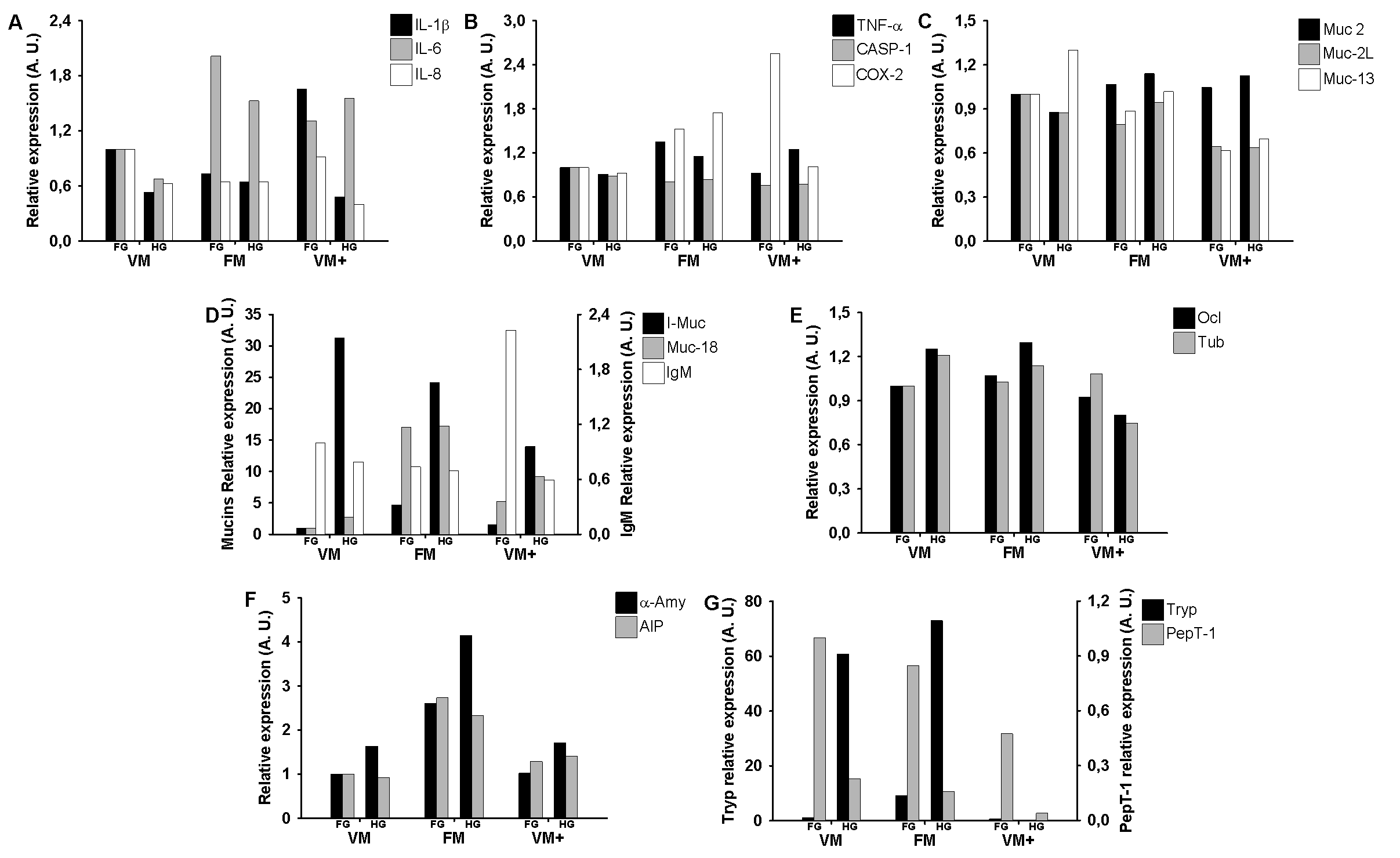

Supplement: Supplementary file 2 — Relative gene expression of candidate target genes in cDNA pooled samples. A) Interleukine-1β (il1β), Interleukin-6 (il6) and Interleukine-8 (il8); B) Tumor Necrosis Factor–α (tnfα), Caspase 1 (casp1), Cyclooxigenase-2 (cox2); C) Mucin 2 (muc2), Mucin 2-like (muc2L), Mucin 13 (muc13); D) Intestinal Mucin (imuc), Mucin 18 (muc18), Immunoglobulin M (igm); E) Occludin (ocl) and Tubuline (tub); F) α-Amylase (αamy) and Alkaline Phosphatase (alp); G) Trypsin (tryp) and Peptide Transporter 1 (pept1). Different genes are represented with different colours. Bars represent relative gene expression of cDNA pools (one per section and treatment), in the foregut (FG) and the hindgut (HG). cDNA pool of the foregut of fish fed VM was used as a calibrator. (TIF 169 kb) [file 12917_2018_1626_MOESM2_ESM.tif]
